# Characterization of Torquetenovirus in amniotic fluid at the time of *in utero* fetal surgery: correlation with early premature delivery and respiratory distress

**DOI:** 10.3389/fmed.2023.1161091

**Published:** 2023-07-20

**Authors:** Tania Regina Tozetto-Mendoza, A. Charlys da- Costa, Antonio F. Moron, Élcio Leal, Silvia Helena Lima, Noely Evangelista Ferreira, Layla Honorato, Heuder Gustavo Oliveira Paião, Wilton Santos Freire, Maria Cássia Mendes-Correa, Steven S. Witkin

**Affiliations:** ^1^Laboratório de Investigação Médica em Virologia (LIM 52), Faculdade de Medicina da Universidade de São Paulo—Instituto de Medicina Tropical de São Paulo, São Paulo, Brazil; ^2^Department of Obstetrics, Universidade Federal de São Paulo, São Paulo, Brazil; ^3^Hospital e Maternidade Santa Joana, São Paulo, Brazil; ^4^Laboratório de Diversidade Viral, Instituto de Ciências Biológicas, Universidade Federal do Pará, Belém, Pará, Brazil; ^5^Departamento de Moléstias Infecciosas e Parasitárias, Universidade de São Paulo, Faculdade de Medicina, São Paulo, Brazil; ^6^Department of Obstetrics and Gynecology, Weill Cornell Medicine, New York, NY, United States

**Keywords:** amniotic fluid, fetal surgery, preterm birth, respiratory distress, spina bifida, Torquetenovirus

## Abstract

Torquetenovirus (TTV) is a commensal virus present in many healthy individuals. Although considered to be non-pathogenic, its presence and titer have been shown to be indicative of altered immune status in individuals with chronic infections or following allogeneic transplantations. We evaluated if TTV was present in amniotic fluid (AF) at the time of *in utero* surgery to correct a fetal neurological defect, and whether its detection was predictive of adverse post-surgical parameters. AF was collected from 27 women by needle aspiration prior to a uterine incision. TTV titer in the AF was measured by isolation of viral DNA followed by gene amplification and analysis. The TTV genomes were further characterized and sequenced by metagenomics. Pregnancy outcome parameters were subsequently obtained by chart review. Three of the AFs (11.1%) were positive for TTV at 3.36, 4.16, and 4.19 log_10_ copies/mL. Analysis of their genomes revealed DNA sequences similar to previously identified TTV isolates. Mean gestational age at delivery was >2  weeks earlier (32.5 vs. 34.6  weeks) and the prevalence of respiratory distress was greater (100% vs. 20.8%) in the TTV-positive pregnancies. TTV detection in AF prior to intrauterine surgery may indicate elevated post-surgical risk for earlier delivery and newborn respiratory distress.

## 1. Introduction

Torquetenovirus (TTV), a small single-strand DNA virus and a member of the *Alphatorquevirus* genus and the *Anelloviridae* family (1), is present at multiple body sites in many healthy individuals. TTV has been detected in blood, feces, saliva and vaginal and cervical secretions (2–6). Only a single study has reported the identification of TTV DNA in amniotic fluid (AF) obtained from six women with uncomplicated pregnancies, and the virus was not isolated or further analyzed (7). While generally considered to be non-pathogenic, multiple studies on patients with chronic infections or who have undergone allogeneic transplantations have demonstrated that the TTV titer is a sensitive indicator of altered immune status (8–10). In these studies, the TTV level is consistently elevated presumably due to a decreased capacity to inhibit their proliferation in immunosuppressed individuals and to a chronic inflammation in those with prolonged infections.

Fetal neural defects are the result of insufficient neurulation of the spinal column during the first month of gestation. Myelomeningocele is a neural tube defect characterized by incomplete closure of the spinal column resulting in protrusion of a sac containing part of the spinal cord, cerebrospinal fluid, meninges and nerves. Rachischisis is a second type of neural tube defect due to a failure of the neural arches and neural tube to fuse leading to external exposure of neural tissue. An encephalocele is characterized by a failure of neural tube closure resulting in the protrusion through the skull of a sac containing brain tissue and spinal fluid. The exposure of neural tissue leads to a range of endocrinological, orthopedic, intellectual and psycho-social problems (11–13). These defects can now be corrected by fetal surgery, in which the defect is closed and the underlying neural tissue is no longer exposed. While outcome results are generally superior to surgery performed following delivery, intrauterine surgery is associated with an elevated rate of spontaneous preterm birth (12, 14, 15).

The availability of AF from 27 cases of *in utero* fetal surgery to correct a myelomeningocele, rachischisis or an encephalocele provided us with a unique opportunity to perform an exploratory investigation of the association of TTV detection with outcome parameters. Our aim was to test the hypothesis that the presence of TTV in AF at the time of *in utero* surgery would be associated with an increased risk of subsequent adverse maternal and/or fetal outcomes. We reasoned that since TTV is present in the circulation, vagina and cervix of pregnant women (4–6) its migration into the amniotic cavity might indicate a pre-existing alteration in uterine immune homeostasis. If TTV is indeed a marker of a disturbed intraamniotic milieu, the added insult of a subsequent invasive *in utero* surgery might increase susceptibility to adverse fetal and/or maternal outcomes in these situations.

## 2. Materials and methods

### 2.1. Study population and procedures

The study was composed of 27 women who underwent fetal surgery at Hospital e Maternidade Santa Joana in São Paulo, Brazil to correct a neural defect in their singleton fetus. Inclusion criterion was indication of a fetal neurological defect correctable by *in utero* surgery. Exclusion criteria were the presence of a defect not amenable to fetal surgery, visible blood contamination of AF and the refusal to provide informed consent. At the start of the operation, prior to an intrauterine incision, approximately 20 mL of AF was aseptically withdrawn with a sterile syringe from the exposed uterus of each subject, as previously described (16). The clear fluid, visibly free of blood contamination, was transferred to sterile tubes and frozen at −80°C until tested. Demographic, surgical and pregnancy-related variables were obtained by chart review after completion of the study. The laboratory personnel were blinded to all clinical data.

The study was approved by the institutional review boards of Hospitale e Maternidade Santa Joana and Hospital São Paulo, Escola Paulista de Medicina, Federal University of São Paulo-UNIFESP, approval number CEP 0598-04/27/2016.

### 2.2. TTV quantification

**Figure 1:**
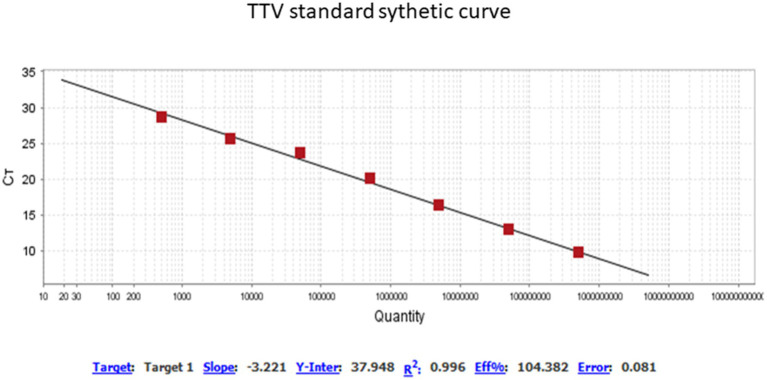
Profile of standard synthetic curve qPCR for TTV quantification. QuantStudio Design & Analysis Software v.1.4.1. Threshold of 0.0271. Titer TTV range of 0.38 to 8.11 log copies/mL.

A thawed AF aliquot from each subject was submitted to DNA extraction using the EasyMag automatized extractor (NucliSENS^®^ easyMag^®^ bioMérieux, Durham, NC), according to the manufacturer’s instructions. All samples were suitable for DNA analysis, i.e., they did not contain inhibitors of DNA amplification, as judged by successful amplification of an internal control (human fragment genic RNAse P) using primers and a probe described previously (17). TTV-specific DNA was amplified using a target to a segment of the untranslated region (UTR) of the viral genome using primers and a probe as previously described by Maggi et al. (18): forward primer 5′-GTGCCGIAGGTGAGTTTA-3′; reverse primer 5′-AGCCCGG CCAGTCC-3′; probe 5′-FAM TCAAGGGGCAATTCGGGCT-MGBNFG 3′. The real time quantitative PCR (qPCR) was performed with 0.25 μM of each primer, 0.062 μM of probe and ~100 ng de DNA template in final volume of 25 μL in TaqMan^®^ Universal Master mix (Thermo Fisher Scientific, Warrington, United Kingdom) according to the manufacturer’s instructions. Thermocycling conditions consisted of two initial heat activation steps of 50°C for 2 min and 95°C for 15 s, followed by 50 cycles of 15 s at 95°C and 1 min at 60°C, in a Quantstudio^™^ 5 instrument. For TTV quantification, the TTV qPCR standard curve was generated using known amounts of HPLC purified synthetic DNA of 73 bp, commercially synthesized (Exxtend Biotecnology Ltda, São Paulo, Brazil): 5′-TTCGTAGCCCGGCCAGTC CCGTATAGCCCGAATTGCCCCTTGAATGCGTTAAACTCACC AI CGGCACCTGATA-3′ as previously described (5). The lyophilized synthetic DNA was diluted in T.E buffer (10 mM Tris, 0.1 mM EDTA, pH 8.0) at initial concentration of 100 pmol/μL, which is approximately 10^13^ DNA molecules/μL, according to Avogadro’s number calculation, and 10-fold serially diluted in nuclease-free water for generating the standard curve, according to the protocols previously described (5, 19). The data were analyzed using QuantStudio Design & Analysis Software v.1.4.1. The analytical sensitivity of TTV qPCR was 40 copies/mL (1.6 log_10_ copies/mL) at ≥95% detection rate. The curve is presented in Figure 1. The qPCR efficiency was of 104.4% (*R*^2^ = 0.99 and slope = −3.22). The parameters of the test quality were based on guidelines previously described (20).

### 2.3. TTV sequencing

The procedure used to perform deep sequencing is a combination of several previously described protocols that have been applied to viral metagenomics and/or virus discovery (21). Briefly, 500uL of AF was centrifuged at 12,000 × g for 10 min, and approximately 400 μL of the supernatant was percolated through a 0.45 μm filter (Merck Millipore, Billerica, MA, United States) to remove eukaryotic and bacterial cell-sized particles. Approximately 100 μL of cold PEG-it Virus Precipitation Solution (System Biosciences, CA, United States) was added to the filtrate, and the contents of the tube was gently mixed and then incubated at 4°C for 24 h. The mixture was then centrifuged at 10,000 × g for 30 min at 4°C and the supernatant (~300 μL) was discarded. The viral particle-rich pellet was treated with a combination of nuclease enzymes (TURBO DNase and RNase Cocktail Enzyme Mix-Thermo Fischer Scientific, CA, United States; Baseline-ZERO DNase-Epicentre, WI, United States; Benzonase-Darmstadt, Germany; and RQ1 RNase Free DNase and RNase A Solution-Promega, WI, United States) to digest unprotected nucleic acids. The resulting mixture was incubated at 37°C for 1 h. Subsequently, viral nucleic acids were extracted using Maxwell^®^ 16 Viral Total Nucleic Acid Purification Kit (Promega, WI, United States), according to the manufacturer’s protocol.

For enrichment of viral DNA the nucleic acid was subjected to rolling circle amplification (RCA) using the TempliPhi 500 Amplification Kit (Cytiva, CA, United States). Briefly, 1 μL of nucleic acid extract plus 5 μL of the kit sample buffer was heated to 95°C for 3 min, cooled on ice, and 5 μL of the kit reaction buffer and 0.2 μL of enzyme mix (Phi29 DNA polymerase) was added. The RCA reaction was incubated at 30°C for 24 h, followed by heat-inactivation at 65°C for 10 min.

Subsequently, a Nextera XT Sample Preparation Kit (Illumina, CA, United States) was used to construct a DNA library, identified using dual barcodes. For size range selection, Pippin Prep (Sage Science, Inc.) was used to select a 600 base pair (bp) insert (range 500–700 bp). The library was deep-sequenced using the NovaSeq 6000 Sequencer (Illumina, CA, United States) with 250 bp ends. Bioinformatics analysis was performed according to the protocol described by Deng et al. (22). Contigs that shared a percent nucleotide identity of 95% or less were assembled from the obtained sequence reads by *de novo* assembly. Based on the bioinformatics pipeline used (22), no reads related to human, plant, fungal, or bacterial sequences were obtained. The final near complete genomes were mapped with Geneious R9 (23).

### 2.4. Alignments and annotation

The resulting contigs were subjected to a modified protein blast search using Ugene software (24) to identify cognate viruses. Based on the best results (best hits) from the BLASTx search, genomes of TTV were chosen for further analysis. Next, full or nearly full genomes were aligned using MAFFT software (25). Genome annotation was performed using GATU software (26), and the TTV sequences OL694814, OL694836, and MN767632 were used as references.

### 2.5. Genetic variability

The maximum composite likelihood model with gamma correction and bootstrap with 100 repetitions was used to calculate nucleotide distances and its standard error. The MEGA software (Version X) was used to calculate distances (27).

### 2.6. Phylogenetic analysis

Phylogenetic trees of TTV genomes were constructed using the maximum likelihood approach. In order to obtain reproducible results and provide greater reliability of the clustering pattern of trees, the statistical support of branches was evaluated by bootstrap approach using 1,000 replications. Trees were inferred using the FastTree (28) software and the GTR model, plus gamma distribution and the proportion of invariable sites were used. Selection of the best model was made according to the likelihood ratio test (LRT) implemented in the Modeltest software (29).

### 2.7. Statistics

Differences between continuous variables were analyzed by the Mann–Whitney test and for discrete variables by the student *t*-test. A two-sided *p*-value <0.05 was considered significant. GraphPad Prism version 8.0.0 for Windows, GraphPad Software, San Diego, California United States, was used for the statistical analysis.

## 3. Results

TTV was detected in 3 (11.1%) of the AF samples. The concentrations of TTV were 3.36, 4.16, and 4.19 log_10_ copies/mL, respectively. Re-analysis of a separate aliquot of TTV-positive or -negative AF always yielded the same result. Similarly, outcomes for positive and negative control samples were always consistent.

Demographic, surgical and maternal and neonatal outcome variables from women with or without TTV in AF are compared in Table 1. TTV was identified in one of two women (50%) whose fetus had an encephalocele, one of 19 women (5.3%) with a fetus with a myelomeningocele and in one of six women (22.2%) carrying a fetus with rachischisis. All three TTV-positive AFs had male fetuses while 50% of the TTV-negative cases were also male. Only one woman, with a TTV-positive AF, was a smoker. The TTV-positive and negative groups were comparable in age, gravidity, parity, length of surgery and length of hospital stay. Of the five women who were non-white, two were positive for TTV; one of the 22 white women had detectable TTV in their AF. As shown in Table 1, the mean gestational age at delivery was 2 weeks earlier (32.5 weeks versus 34.4 weeks) in women with a TTV-positive AF (*p* = 0.0388), and the mean neonatal birthweight (1995 g vs. 2,450 g) was reduced when TTV was present in the AF (*p* = 0.0769).

**Table 1:** Study population characteristics.

Characteristic	TTV-negative *N* = 24	TTV positive *N* = 3	*p*
Mean age (SD)	32.6 (3.9) years	33.0 (3.6) years	
Median gravidity (range)	2 (1–3)	2 (1–5)	
Median parity (range)	1 (0–2)	1 (0–2)	
*Race*			
White	21 (87.5%)	1 (33.3%)	
Non-white	3 (12.5%)	2 (66.7%)	
Mean gestational age at surgery (SD)	25.1 (0.8) years	25.3 (1.2) years	
Mean length of surgery (SD)	125 (22) minutes	155 (32) minutes	
Mean time for wound healing (SD)	7.2 (2.0) days	6.7 (0.6) days	
Mean hospital stay (SD)	7.5 (3.1) days	6.0 (0) days	
Mean gestational age at delivery (SD)	34.4 (2.1) weeks	32.5 (0.5) weeks	0.0388
Male infant	12 (50%)	3 (100%)	
Smoker	0	1 (33.3%)	
Mean birthweight (SD)	2450 (460) g	1995 (163) g	0.0769
Respiratory distress of newborn	5 (20.8%)	3 (100%)	0.0191
*Type of spinal lesion*			
Myelomeningocele	18 (75%)	1 (33.3%)	
Rachischisis	5 (20.8%)	1 (33.3%)	
Encephalocele	1 (4.2%)	1 (33.3%)	

**Figure 2:**
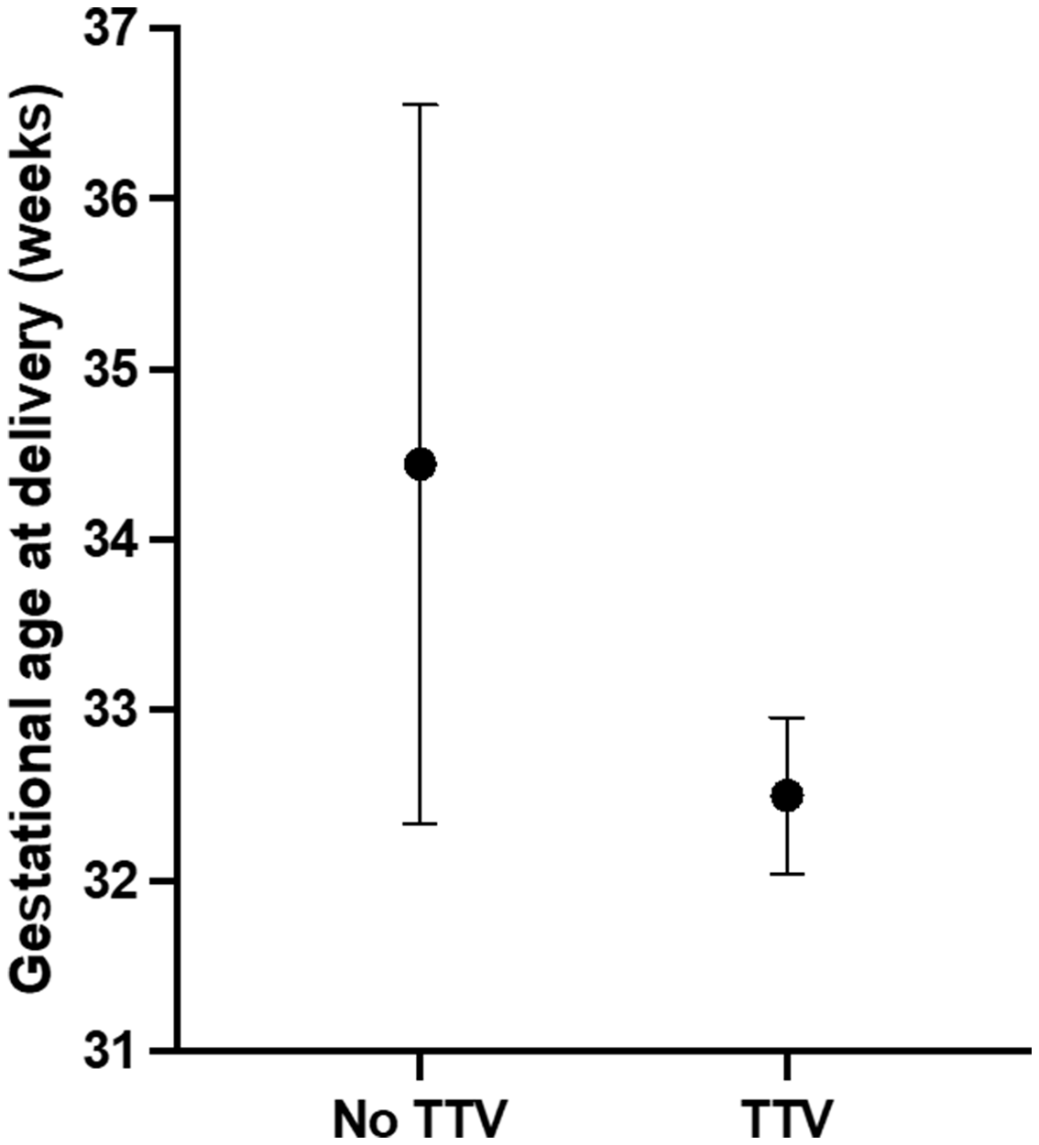
Gestational age at delivery in pregnancies positive or negative for TTV in amniotic fluid. TTV in amniotic fluid was detected by gene amplification. Pregnancy outcome was subsequently obtained by chart review. Values are expressed as mean and 95% confidence intervals. The dot indicates the mean value.

The distribution of deliveries in each group is shown in Figure 2. In addition, all three (100%) of the neonates from TTV-positive AF experienced respiratory distress, as opposed to 5 (20.8%) of the 24 neonates from AFs that were negative for TTV (*p* = 0.0191).

Characterization of the three TTV sequences is described in Table 2. The sequences have been deposited in GenBank under accession numbers OP882564, OP882565, and OP882567. Following generation of the consensus sequences TTV was detected in the three positive AFs with 980×, 630× and 8,956× coverage, respectively. The length of the TTV genome detected was 3,558, 3,889 and 2,680 bp, respectively. This corresponded to 96, 100 and 100%, respectively, of the total TTV genome covered in relation to the references TTV genomes used. TTV accession number OP882565 was 100% identical to TTV6 first isolated from the blood of a subject in The Netherlands in 2022 (accession number OL694836.1). Our TTV OP882564 was 99% identical to an unclassified TTV isolated from febrile children in Tanzania (accession number MN767632.1). Our TTV OP882567 was 99% identical to a different unclassified TTV identified in serum from febrile children in Tanzania in 2015 (accession number MN767455.1).

**Table 2:** Characterization of the TTV isolates.

GenBank Accession No.	Mapped reads	Genome length	% TTV genome covered	% identity with known TTV isolates
OP882565	4,910	3,889	100	100
OP882564	6,985	3,558	96	99
OP882567	48,004	2,680	100	99

**Figure 3:**
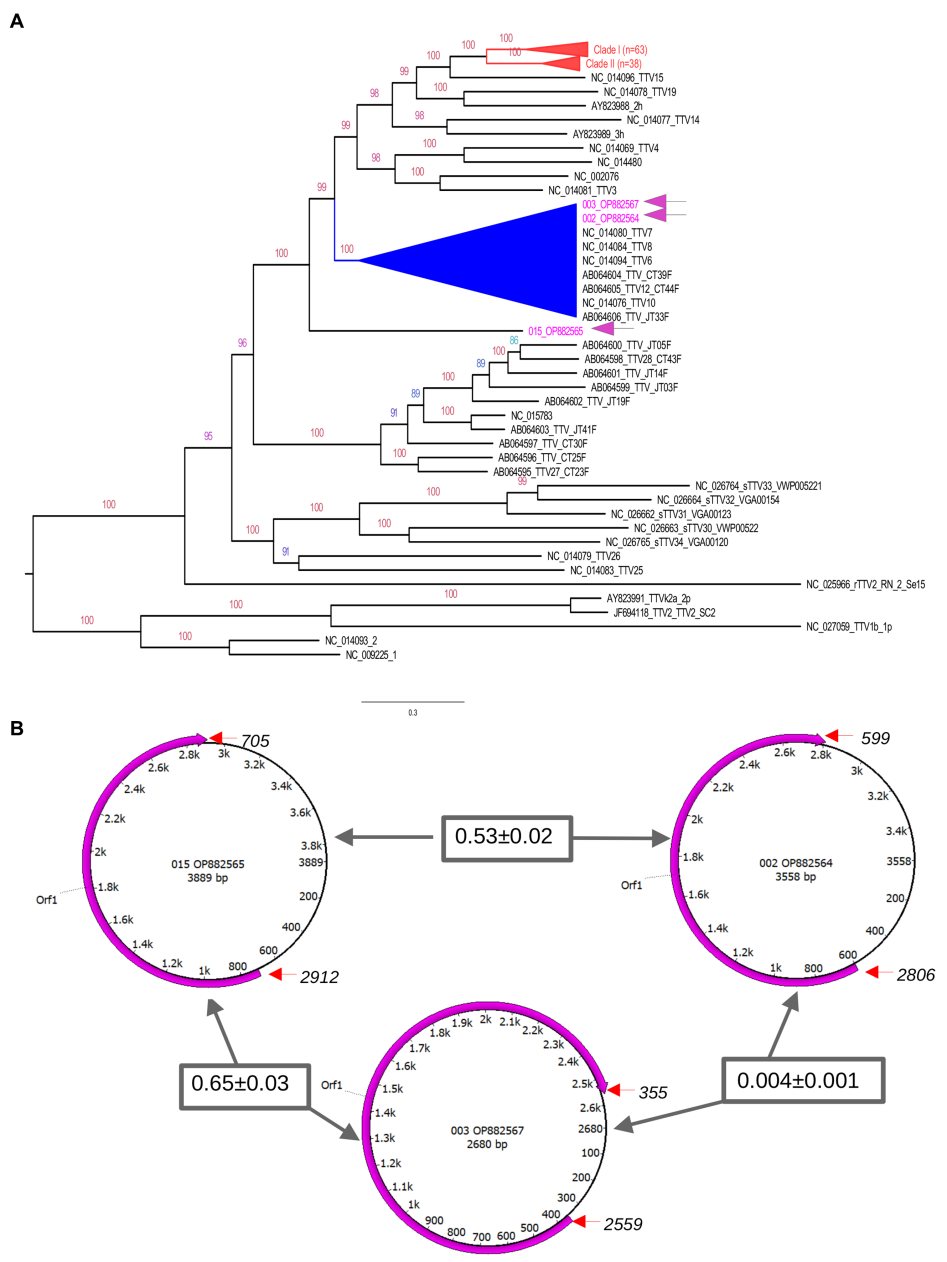
Genetic features of TTV genomes. The upper panel **(A)** is the phylogenetic tree inferred using complete genomes of TTV. The tree indicates that there are multiple clades (indicated by distinct colors) composed by TTV identified in different countries. Sequences generated in this study are indicated in blue in the tree. Number at nodes are bootstrap values. Some clusters were collapsed to facilitate visualization (indicated in red). Clades with high bootstrap values are indicated in different colors. All sequences identified in AF from Brazilian patients grouped in a distinct cluster. The exception is the sequence OP882566 located in a unique branch. The lower panel **(B)** shows the genomic annotation of TTV identified in the current study. Circles represent the single stranded circular genome of TTV. Numbers within these circles indicate the nucleotide position and size. The magenta semi-circular arrows show the position of the predicted ORF1 based on TTV references. The start and end of orf1 are indicated by red arrows. Numbers inside rectangles are the nucleotide divergence of the ORF1 gene region between the TTV sequences.

To construct a phylogenetic tree, we included 140 known TTV sequences as references. These sequences were collected from GenBank based on similarity to our sequences after the initial BlastN search. The tree was constructed by a maximum likelihood approach using GTR model and gamma correction (Figure 3A). The topology shows clusters of sequences with high branch support (bootstrap values higher than 90). These clusters included sequences identified mostly in humans from different geographical regions. It is important to note that our sequences clustered in divergent phyloclades. One sequence (OP882565) formed a unique branch at the base of the above-mentioned clusters. The genomic annotation indicated that although our sequences are divergent their genomic maps are similar (Figure 3B). We used the references OL694836, OL694814, and MN767632 to annotated our sequences OP882564, OP882565, and OP882567, respectively. One single open reading frame (ORF1) was detected, located between nucleotides 750 to 3,035 in OP882564; between 705 to 2,912 in OP882565 and between nucleotides 355 to 2,559 in the sequence OP882567. The genetic diversity was estimated using complete genomes and indicated that the nucleotide distance between OP882567 and OP882565 is 77% (SD ± 3%), between OP882567 and OP882564 is 77% (SD ± 3%) and between OP882565 and OP882564 is 82% (SD ± 3%).

## 4. Discussion

TTV was identified in AF from 3 women who were undergoing *in utero* fetal surgery to correct a neural tube defect in their fetus. The nearly complete DNA sequences generated from each TTV isolate was similar to that of known TTV genomes. The collection of AF from the exposed uterus before intrauterine surgery negated the possibility of maternal blood contamination of the samples. The results demonstrate that TTV can be detected in AF from some women carrying a fetus with a neurological defect. The association of AF TTV with lower gestational age at delivery (*p* = 0.0388) and the occurrence of neonatal respiratory distress following surgery (*p* = 0.0191) suggests that its presence in AF prior to surgery is a potential biomarker for increased susceptibility to post-surgical adverse pregnancy outcomes. The involved mechanism remains to be determined.

AF is composed of fetal urine and pulmonary secretions, transudation of compounds from the maternal circulation and secretions from amniotic cells (30). In addition, it is likely that the AF from women with fetuses with neurological defects differ from AF of healthy normal pregnancies due to exposure of the meninges and leakage of cerebrospinal fluid. Whether this leakage was the source of TTV and/or created a unique environment permissive for TTV proliferation, remains to be determined. It is also presently unknown, and worthy of study, if TTV detection in AF from women carrying a neurologically normal fetus might also be associated with elevated risk for adverse outcomes.

The detection of distinct lineages of TTV in AF from only three of 27 cases with these anomalies suggests that this virus may not be a major teratogenic influence on fetal neurological development. Although TTV DNA load in the serum fluctuates under different pathological conditions, there is no evidence that this virus is the causative agent of any disease (31). Moreover, it has been proposed that TTV is a plasmid-like element without capsid, although the expression of TTV proteins has been demonstrated *in vitro* (32). However, a much larger study will be required to detect the possible occurrence of more subtle TTV-involved differences in these fetuses. Similarly, whether TTV is preferentially present in AF from non-white women needs further verification.

A strength of the study is that all surgeries were performed by the same team with a high degree of experience in the procedure (16). A limitation of the study is that there are undoubtedly multiple mechanisms leading to early preterm birth and neonatal morbidity. TTV detection may be indicative of only one such mechanism. The present study did not include the use of positive and negative controls for DNA of other viruses, that may have also possibly been present. We also cannot rule out the possibility that our primers/probe set designed for a conserved UTR region of the TTV genome were restricted, since studies of TTV genome sequences have indicated that a qPCR with primers and probe may fail to detect some TTV strains (33). The obvious major limitation to any interpretation is that only 27 samples were available for analysis and the detection of only three TTV-positive AF samples. However, this study population is unique and relatively rare. The findings, therefore, must be designated as exploratory. However, in our opinion the observations in this communication are novel, worthy of transmission and of sufficient high potential interest to encourage further investigation.

## 5. Conclusion

Alterations in intra-amniotic conditions conducive to the appearance and possible proliferation of TTV in AF from pregnant women carrying a fetus with a neurological defect may signal an elevated susceptibility to adverse post- surgical outcome.

## Data availability statement

The datasets presented in this study can be found in online repositories. The names of the repository/repositories and accession number(s) can be found at: https://www.ncbi.nlm.nih.gov/genbank/, OP882565 https://www.ncbi.nlm.nih.gov/genbank/, OP882564 https://www.ncbi.nlm.nih.gov/genbank/, OP882567.

## Ethics statement

The studies involving human participants were reviewed and approved by the institutional review boards of Hospitale e Maternidade Santa Joana and Hospital São Paulo, Escola Paulista de Medicina, Federal University of São Paulo-UNIFESP, approval number CEP 0598-04/27/2016. The patients/participants provided their written informed consent to participate in this study.

## Author contributions

TT-M, WF, and LH developed, performed and interpreted the quantitative qPCR TTV detection analysis. SL, NF, and HP performed the DNA isolation method and data analysis. Ad-C and ÉL isolated TTV from amniotic fluid by metagenomics and characterized the viral genomes. AM supervised all surgical and clinical aspects of the study and collected the amniotic fluid. MM-C supervised all aspects of the laboratory investigation. SW conceived of the study, supervised data analysis, and wrote the initial draft of the manuscript. All authors contributed to the article and approved the submitted version.

## Funding

The study was funded by specific grants from *laboratório de Investigação Médica em Virologia*—*LIM 52*.

## Conflict of interest

The authors declare that the research was conducted in the absence of any commercial or financial relationships that could be construed as a potential conflict of interest.

## Publisher’s note

All claims expressed in this article are solely those of the authors and do not necessarily represent those of their affiliated organizations, or those of the publisher, the editors and the reviewers. Any product that may be evaluated in this article, or claim that may be made by its manufacturer, is not guaranteed or endorsed by the publisher.
